# 8-Formyl-4-methyl-2-oxo-2*H*-chromen-7-yl 4-methyl­benzenesulfonate

**DOI:** 10.1107/S1600536811018927

**Published:** 2011-05-25

**Authors:** H. Yuvaraj, D. Gayathri, Rajesh G. Kalkhambkar, Geeta M. Kulkarni, Rajendra M. Bapset

**Affiliations:** a School of Display and Chemical Engineering, Yeungnam University, Gyeongsan, Gyeoungbuk 712-749, Republic of Korea; b Department of Physics, Dr. M.G.R. Educational and Research Institute University, Periyar E.V.R. High Road, Maduravoyal, Chennai 600 095, India; c Department of Chemistry, Karnatak University’s Karnatak Science College, Dharwad 580 001, Karnataka, India; d Department of Chemistry, B.K. College, Belgaum 590 001, Karnataka, India

## Abstract

In the title compound, C_18_H_14_O_6_S, the coumarin ring system is nearly planar, with a maximum out-of-plane deviation of 0.032 (2) Å. The dihedral angle between the benzene ring and the coumarin ring system is 32.41 (8)°. The crystal packing is stabilized by inter­molecular C—H⋯O inter­actions, generating *C*(8), *C*(10) and *C*(11) chains and an *R*_2_^2^(10) ring. The formyl group is disordered over two sets of sites, with occupancies of 0.548 (5) and 0.452 (5).

## Related literature

For the biological activity of coumarins, see: Carlton *et al.* (1996 ▶); El-Agrody *et al.* (2001 ▶); Emmanuel-Giota *et al.* (2001 ▶); Kulkarni *et al.* (2006 ▶); Kalkhambkar *et al.* (2008 ▶); Shaker (1996 ▶); Yang *et al.* (2005 ▶); Zhou *et al.* (2000 ▶). For a related structure, see: Yuvaraj *et al.* (2011 ▶).
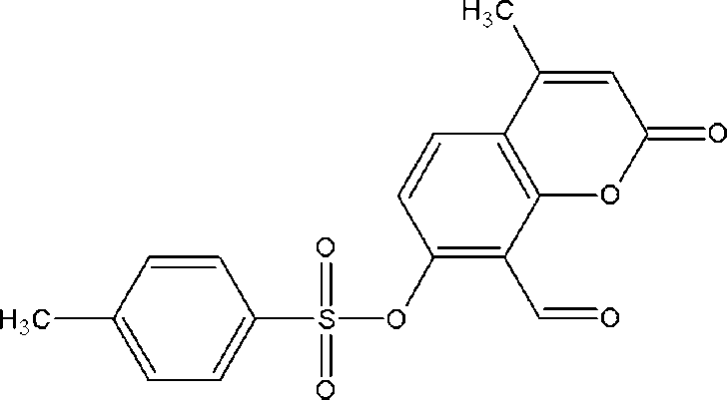


## Experimental

### 

#### Crystal data

C_18_H_14_O_6_S
*M* *_r_* = 358.35
Orthorhombic, 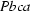

*a* = 17.6174 (7) Å
*b* = 7.2025 (3) Å
*c* = 25.7706 (10) Å
*V* = 3270.0 (2) Å^3^
*Z* = 8
Mo *K*α radiation
μ = 0.23 mm^−1^
*T* = 293 K
0.14 × 0.13 × 0.13 mm

#### Data collection

Bruker SMART APEX CCD area-detector diffractometer
48911 measured reflections
4234 independent reflections
3095 reflections with *I* > 2σ(*I*)
*R*_int_ = 0.037

#### Refinement

*R*[*F* ^2^ > 2σ(*F* ^2^)] = 0.043
*wR*(*F* ^2^) = 0.135
*S* = 1.02
4234 reflections
238 parameters
14 restraints
H-atom parameters constrained
Δρ_max_ = 0.26 e Å^−3^
Δρ_min_ = −0.37 e Å^−3^

### 

Data collection: *SMART* (Bruker, 2001 ▶); cell refinement: *SAINT* (Bruker, 2001 ▶); data reduction: *SAINT*; program(s) used to solve structure: *SHELXS97* (Sheldrick, 2008 ▶); program(s) used to refine structure: *SHELXL97* (Sheldrick, 2008 ▶); molecular graphics: *PLATON* (Spek, 2009 ▶); software used to prepare material for publication: *WinGX* (Farrugia, 1999 ▶) and *PARST* (Nardelli, 1995 ▶).

## Supplementary Material

Crystal structure: contains datablocks I, global. DOI: 10.1107/S1600536811018927/is2704sup1.cif

Structure factors: contains datablocks I. DOI: 10.1107/S1600536811018927/is2704Isup2.hkl

Supplementary material file. DOI: 10.1107/S1600536811018927/is2704Isup3.cml

Additional supplementary materials:  crystallographic information; 3D view; checkCIF report

## Figures and Tables

**Table 1:** Hydrogen-bond geometry (Å, °)

*D*—H⋯*A*	*D*—H	H⋯*A*	*D*⋯*A*	*D*—H⋯*A*
C4—H4⋯O6^i^	0.93	2.56	3.326 (2)	140
C13—H13⋯O6^ii^	0.93	2.52	3.343 (2)	148
C16—H16⋯O2^iii^	0.93	2.51	3.433 (2)	175

Symmetry codes: (i) 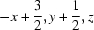; (ii) 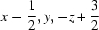; (iii) 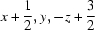.

**Fig. 1.**
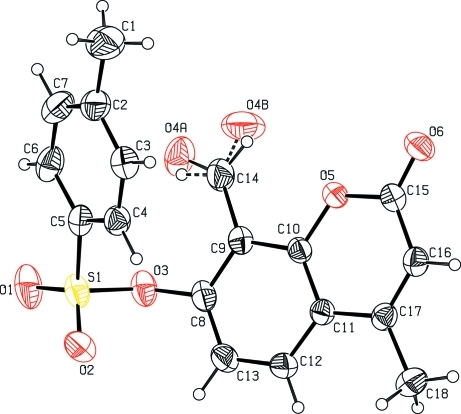
The molecular structure of the title compound, showing 30% probability displacement ellipsoids.

**Fig. 2.**
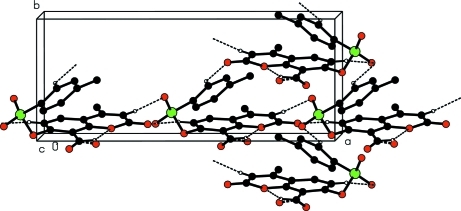
The molecular packing of the title compound, showing the C—H···O interactions (dashed lines). For clarity, hydrogen atoms which are not involved in hydrogen bonding are omitted.

## supplementary crystallographic information

### Comment

Coumarins are the class of lactones with extensive occurrence in plants possessing wide range of biological activities (Kulkarni *et al.*, 2006). Many synthetic and naturally occurring coumarins are well documented for their wide range of biological activities such as antibacterial (El-Agrody *et al.*, 2001), antifungal (Shaker, 1996), antioxidant (Yang *et al.*, 2005), analgesic (Kalkhambkar *et al.*, 2008) and anti-inflammatory (Emmanuel-Giota *et al.*, 2001) properties. A large number of natural and semisynthetic coumarin and bicoumarin derivatives have been reported to demonstrate chemopreventive (Carlton *et al.*, 1996) and anti-HIV (Zhou *et al.*, 2000) activities. In view of various biological properties associated with coumarins, we have synthesized the title compound and report here its structure.

The molecular structure of the title compound is shown in Fig.1. Bond lengths and bond angles are comparable with the similar structure (Yuvaraj *et al.*, 2011). Positional disorder has been observed for formyl oxygen atom with C14—O4A and C14—O4B bond lengths being 1.137 (3) and 1.109 (4) Å, respectively. The coumarin ring system is nearly planar with a maximum out-of-plane deviation of 0.032 (2) Å (r.m.s. deviation = 0.021 Å). Dihedral angle between the C2–C7 benzene ring and the C8–C13 benzene ring of the coumarin moeity is 33.3 (1)°. Atoms O6 and C18 lie -0.017 (3) and 0.073 (3) Å, respectively, from the least-squares plane of the atoms (C10/C11/C17/C16/C15/O5, r.m.s. deviation = 0.012 Å). Atom C1 lies 0.030 (4) Å from the least-squares plane of the phenyl ring (r.m.s. deviation = 0.007 Å).

The crystal packing is stabilized by C—H···O intermolecular interactions. C4—H4···O6^i^ interaction generates a helical C(11) chain along the [010] direction. Interactions C13—H13···O6^ii^ and C16—H16···O2^iii^ generate C(8) and C(10) chains, respectively, running along [100]. In addition, the interactions C13—H13···O6^ii^ and C16—H16···O2^iii^ generate an *R*_2_^2^(10) ring.

### Experimental

A mixture of 8-formyl-7-hydroxy-4-methyl coumarin (6 mmol), *p*-toluenesulfonylchloride (6 mmol) and powdered anhydrous K_2_CO_3_ (6 mmol) in 20 ml of super dry acetone was stirred in room temperature for 12 h. After the completion of the reaction, the solvent was removed under reduced pressure and separated the solids by filtration. It was then washed with 50 ml of dilute hydrochloric acid, excess of cold water, dried and crystallized from ethanol and dioxan mixture. Yield 80%; Light green crystalline solid (ethanol + dioxan); m.p. 180–182 °C; *R*_f_ 0.43 (benzene); IR (KBr) cm^-1^ 1734, 1708, 1303; ^1^H NMR (CDCl_3_) δ 2.47 (3*H*, s), 2.51 (3*H*, s), 6.35 (1*H*, s), 7.32 (6*H*, m), 10.34 (1*H*, s); Anal. Calcd. for C_18_H_14_O_6_S: C 60.33, H 3.94; Found: C 60.13, H 3.70.

### Refinement

All H-atoms were refined using a riding model, with *d*(C—H) = 0.93 Å and *U*_iso_(H) = 1.2*U*_eq_(C) for the aromatic CH, and with *d*(C—H) = 0.96 Å and *U*_iso_(H) = 1.5*U*_eq_(C) for the CH_3_ group. The formyl group is disordered over two sites with occupancies of 0.548 (5) and 0.452 (5). Atoms O4A and O4B were restrained to be approximately isotropic by an *ISOR* 0.01 command. The distances of C14—O4A and C14—O4B have been restrained to 1.20 (1) Å.

### Crystal data

**Table:**

C_18_H_14_O_6_S	*F*(000) = 1488
*M**_r_* = 358.35	*D*_x_ = 1.456 Mg m^−^^3^
Orthorhombic, *P**b**c**a*	Mo *K*α radiation, λ = 0.71073 Å
Hall symbol: -P 2ac 2ab	Cell parameters from 6552 reflections
*a* = 17.6174 (7) Å	θ = 2.3–26.6°
*b* = 7.2025 (3) Å	µ = 0.23 mm^−^^1^
*c* = 25.7706 (10) Å	*T* = 293 K
*V* = 3270.0 (2) Å^3^	Plate, colorless
*Z* = 8	0.14 × 0.13 × 0.13 mm

### Data collection

**Table:**

Bruker SMART APEX CCD area-detector diffractometer	3095 reflections with *I* > 2σ(*I*)
Radiation source: fine-focus sealed tube	*R*_int_ = 0.037
graphite	θ_max_ = 28.7°, θ_min_ = 1.6°
φ and ω scans	*h* = −23→23
48911 measured reflections	*k* = −9→9
4234 independent reflections	*l* = −34→34

### Refinement

**Table:**

Refinement on *F*^2^	Primary atom site location: structure-invariant direct methods
Least-squares matrix: full	Secondary atom site location: difference Fourier map
*R*[*F*^2^ > 2σ(*F*^2^)] = 0.043	Hydrogen site location: inferred from neighbouring sites
*wR*(*F*^2^) = 0.135	H-atom parameters constrained
*S* = 1.02	*w* = 1/[σ^2^(*F*_o_^2^) + (0.0672*P*)^2^ + 0.8242*P*] where *P* = (*F*_o_^2^ + 2*F*_c_^2^)/3
4234 reflections	(Δ/σ)_max_ = 0.001
238 parameters	Δρ_max_ = 0.26 e Å^−^^3^
14 restraints	Δρ_min_ = −0.37 e Å^−^^3^

### Special details

**Table:**

Geometry. All e.s.d.'s (except the e.s.d. in the dihedral angle between two l.s. planes) are estimated using the full covariance matrix. The cell e.s.d.'s are taken into account individually in the estimation of e.s.d.'s in distances, angles and torsion angles; correlations between e.s.d.'s in cell parameters are only used when they are defined by crystal symmetry. An approximate (isotropic) treatment of cell e.s.d.'s is used for estimating e.s.d.'s involving l.s. planes.
Refinement. Refinement of *F*^2^ against ALL reflections. The weighted *R*-factor *wR* and goodness of fit *S* are based on *F*^2^, conventional *R*-factors *R* are based on *F*, with *F* set to zero for negative *F*^2^. The threshold expression of *F*^2^ > σ(*F*^2^) is used only for calculating *R*-factors(gt) *etc*. and is not relevant to the choice of reflections for refinement. *R*-factors based on *F*^2^ are statistically about twice as large as those based on *F*, and *R*- factors based on ALL data will be even larger.

### Fractional atomic coordinates and isotropic or equivalent isotropic displacement parameters (Å^2^)

**Table:**

	*x*	*y*	*z*	*U*_iso_*/*U*_eq_	Occ. (<1)
C1	0.70245 (18)	0.5004 (5)	0.48070 (11)	0.1076 (9)	
H1A	0.7160	0.4100	0.4550	0.161*	
H1B	0.7446	0.5202	0.5037	0.161*	
H1C	0.6895	0.6152	0.4639	0.161*	
C2	0.63513 (13)	0.4313 (3)	0.51137 (8)	0.0712 (5)	
C3	0.62555 (11)	0.4882 (3)	0.56260 (8)	0.0652 (5)	
H3	0.6605	0.5692	0.5773	0.078*	
C4	0.56508 (10)	0.4264 (3)	0.59184 (7)	0.0585 (4)	
H4	0.5587	0.4663	0.6259	0.070*	
C5	0.51398 (10)	0.3038 (2)	0.56962 (7)	0.0563 (4)	
C6	0.52174 (14)	0.2479 (3)	0.51834 (8)	0.0708 (5)	
H6	0.4866	0.1677	0.5034	0.085*	
C7	0.58218 (16)	0.3130 (3)	0.49004 (8)	0.0791 (6)	
H7	0.5875	0.2763	0.4556	0.095*	
C8	0.54138 (10)	0.0811 (3)	0.67455 (7)	0.0578 (4)	
C9	0.61696 (10)	0.0624 (2)	0.65996 (6)	0.0525 (4)	
C10	0.67090 (9)	0.1065 (2)	0.69784 (6)	0.0472 (3)	
C11	0.65136 (9)	0.1600 (2)	0.74813 (6)	0.0495 (4)	
C12	0.57417 (11)	0.1691 (3)	0.76024 (7)	0.0631 (5)	
H12	0.5595	0.2012	0.7937	0.076*	
C13	0.51960 (10)	0.1319 (3)	0.72389 (8)	0.0680 (5)	
H13	0.4684	0.1408	0.7324	0.082*	
C14	0.63965 (14)	−0.0075 (4)	0.60842 (8)	0.0772 (6)	
H14A	0.6916	0.0009	0.6025	0.093*	0.548 (5)
H14B	0.5992	−0.0394	0.5871	0.093*	0.452 (5)
C15	0.80427 (9)	0.1381 (2)	0.71607 (7)	0.0534 (4)	
C16	0.78344 (10)	0.1945 (2)	0.76774 (7)	0.0550 (4)	
H16	0.8220	0.2264	0.7907	0.066*	
C17	0.71185 (10)	0.2035 (2)	0.78441 (6)	0.0513 (4)	
C18	0.69355 (13)	0.2552 (3)	0.83935 (7)	0.0720 (5)	
H18A	0.7398	0.2772	0.8581	0.108*	
H18B	0.6660	0.1558	0.8555	0.108*	
H18C	0.6631	0.3658	0.8396	0.108*	
O1	0.38782 (9)	0.1146 (3)	0.57769 (7)	0.0957 (6)	
O2	0.41980 (9)	0.3388 (2)	0.64607 (6)	0.0814 (4)	
O3	0.48511 (7)	0.03917 (19)	0.63759 (6)	0.0685 (4)	
O4A	0.60724 (19)	−0.0694 (4)	0.57442 (11)	0.0912 (13)	0.548 (5)
O4B	0.6962 (2)	−0.0293 (10)	0.59052 (17)	0.136 (2)	0.452 (5)
O5	0.74534 (6)	0.09179 (18)	0.68306 (4)	0.0539 (3)	
O6	0.86731 (7)	0.1254 (2)	0.69874 (6)	0.0738 (4)	
S1	0.44287 (3)	0.20842 (8)	0.60799 (2)	0.06651 (18)	

### Atomic displacement parameters (Å^2^)

**Table:**

	*U*^11^	*U*^22^	*U*^33^	*U*^12^	*U*^13^	*U*^23^
C1	0.113 (2)	0.114 (2)	0.0953 (18)	0.0111 (18)	0.0263 (16)	0.0339 (16)
C2	0.0815 (14)	0.0662 (12)	0.0660 (11)	0.0140 (10)	0.0039 (10)	0.0138 (10)
C3	0.0622 (11)	0.0601 (10)	0.0735 (12)	0.0003 (9)	−0.0090 (9)	0.0002 (9)
C4	0.0580 (10)	0.0607 (10)	0.0569 (9)	0.0028 (8)	−0.0095 (8)	−0.0079 (8)
C5	0.0581 (9)	0.0538 (9)	0.0570 (9)	0.0043 (8)	−0.0142 (8)	−0.0006 (7)
C6	0.0929 (15)	0.0587 (10)	0.0607 (10)	0.0035 (10)	−0.0224 (10)	−0.0065 (9)
C7	0.1139 (18)	0.0721 (13)	0.0513 (10)	0.0132 (13)	−0.0026 (11)	−0.0005 (9)
C8	0.0468 (8)	0.0596 (10)	0.0671 (10)	−0.0013 (7)	−0.0085 (7)	0.0120 (8)
C9	0.0506 (9)	0.0523 (9)	0.0546 (9)	0.0037 (7)	−0.0045 (7)	0.0034 (7)
C10	0.0425 (8)	0.0477 (8)	0.0513 (8)	0.0046 (6)	−0.0005 (6)	0.0047 (6)
C11	0.0489 (8)	0.0501 (8)	0.0496 (8)	0.0055 (7)	0.0010 (7)	0.0078 (7)
C12	0.0554 (10)	0.0788 (12)	0.0549 (9)	0.0106 (9)	0.0095 (8)	0.0116 (9)
C13	0.0442 (9)	0.0882 (14)	0.0715 (11)	0.0044 (9)	0.0055 (8)	0.0176 (10)
C14	0.0680 (13)	0.0969 (16)	0.0667 (12)	0.0167 (12)	−0.0161 (11)	−0.0185 (11)
C15	0.0473 (8)	0.0543 (9)	0.0588 (9)	0.0035 (7)	−0.0040 (7)	0.0000 (7)
C16	0.0550 (9)	0.0540 (9)	0.0559 (9)	0.0008 (7)	−0.0089 (7)	−0.0021 (7)
C17	0.0589 (9)	0.0468 (8)	0.0483 (8)	0.0035 (7)	−0.0022 (7)	0.0030 (7)
C18	0.0799 (13)	0.0841 (13)	0.0519 (9)	0.0003 (11)	0.0025 (9)	−0.0045 (9)
O1	0.0631 (9)	0.1010 (12)	0.1230 (14)	−0.0170 (8)	−0.0409 (9)	0.0130 (10)
O2	0.0605 (8)	0.0953 (11)	0.0884 (10)	0.0165 (8)	0.0070 (7)	0.0052 (9)
O3	0.0530 (7)	0.0674 (8)	0.0850 (9)	−0.0080 (6)	−0.0181 (6)	0.0112 (7)
O4A	0.111 (3)	0.084 (2)	0.0780 (19)	0.0174 (17)	−0.0341 (17)	−0.0248 (15)
O4B	0.078 (3)	0.241 (6)	0.089 (3)	−0.013 (3)	0.016 (2)	−0.065 (3)
O5	0.0435 (6)	0.0660 (7)	0.0522 (6)	0.0061 (5)	−0.0009 (5)	−0.0045 (5)
O6	0.0454 (7)	0.0988 (11)	0.0773 (9)	0.0045 (7)	0.0020 (6)	−0.0126 (8)
S1	0.0460 (2)	0.0741 (3)	0.0795 (3)	−0.0003 (2)	−0.0156 (2)	0.0070 (2)

### Geometric parameters (Å, °)

**Table:**

C1—C2	1.510 (3)	C11—C12	1.397 (2)
C1—H1A	0.9600	C11—C17	1.452 (2)
C1—H1B	0.9600	C12—C13	1.369 (3)
C1—H1C	0.9600	C12—H12	0.9300
C2—C7	1.378 (3)	C13—H13	0.9300
C2—C3	1.393 (3)	C14—O4B	1.109 (4)
C3—C4	1.379 (3)	C14—O4A	1.137 (3)
C3—H3	0.9300	C14—H14A	0.9300
C4—C5	1.385 (2)	C14—H14B	0.9300
C4—H4	0.9300	C15—O6	1.200 (2)
C5—C6	1.388 (3)	C15—O5	1.383 (2)
C5—S1	1.737 (2)	C15—C16	1.440 (2)
C6—C7	1.373 (3)	C16—C17	1.334 (2)
C6—H6	0.9300	C16—H16	0.9300
C7—H7	0.9300	C17—C18	1.499 (2)
C8—C13	1.378 (3)	C18—H18A	0.9600
C8—C9	1.390 (2)	C18—H18B	0.9600
C8—O3	1.407 (2)	C18—H18C	0.9600
C9—C10	1.399 (2)	O1—S1	1.4165 (16)
C9—C14	1.476 (3)	O2—S1	1.4179 (17)
C10—O5	1.3696 (19)	O3—S1	1.6191 (14)
C10—C11	1.395 (2)		
			
C2—C1—H1A	109.5	C12—C11—C17	124.09 (16)
C2—C1—H1B	109.5	C13—C12—C11	121.44 (17)
H1A—C1—H1B	109.5	C13—C12—H12	119.3
C2—C1—H1C	109.5	C11—C12—H12	119.3
H1A—C1—H1C	109.5	C12—C13—C8	119.21 (17)
H1B—C1—H1C	109.5	C12—C13—H13	120.4
C7—C2—C3	118.6 (2)	C8—C13—H13	120.4
C7—C2—C1	121.8 (2)	O4B—C14—C9	131.8 (3)
C3—C2—C1	119.6 (2)	O4A—C14—C9	133.7 (3)
C4—C3—C2	121.12 (19)	C9—C14—H14A	113.1
C4—C3—H3	119.4	C9—C14—H14B	114.2
C2—C3—H3	119.4	O6—C15—O5	116.59 (16)
C3—C4—C5	118.82 (17)	O6—C15—C16	126.95 (16)
C3—C4—H4	120.6	O5—C15—C16	116.45 (15)
C5—C4—H4	120.6	C17—C16—C15	123.55 (16)
C4—C5—C6	120.96 (19)	C17—C16—H16	118.2
C4—C5—S1	119.03 (14)	C15—C16—H16	118.2
C6—C5—S1	119.89 (16)	C16—C17—C11	118.42 (15)
C7—C6—C5	118.9 (2)	C16—C17—C18	121.31 (17)
C7—C6—H6	120.6	C11—C17—C18	120.27 (16)
C5—C6—H6	120.6	C17—C18—H18A	109.5
C6—C7—C2	121.63 (19)	C17—C18—H18B	109.5
C6—C7—H7	119.2	H18A—C18—H18B	109.5
C2—C7—H7	119.2	C17—C18—H18C	109.5
C13—C8—C9	122.83 (17)	H18A—C18—H18C	109.5
C13—C8—O3	119.04 (16)	H18B—C18—H18C	109.5
C9—C8—O3	118.08 (17)	C8—O3—S1	118.74 (12)
C8—C9—C10	116.10 (16)	C10—O5—C15	121.95 (13)
C8—C9—C14	122.41 (17)	O1—S1—O2	120.07 (11)
C10—C9—C14	121.43 (16)	O1—S1—O3	102.44 (9)
O5—C10—C11	121.05 (14)	O2—S1—O3	107.72 (9)
O5—C10—C9	116.03 (14)	O1—S1—C5	111.62 (10)
C11—C10—C9	122.91 (15)	O2—S1—C5	109.80 (10)
C10—C11—C12	117.44 (16)	O3—S1—C5	103.55 (8)
C10—C11—C17	118.47 (15)		
			
C7—C2—C3—C4	0.8 (3)	C8—C9—C14—O4B	179.2 (6)
C1—C2—C3—C4	−179.4 (2)	C10—C9—C14—O4B	−3.5 (7)
C2—C3—C4—C5	0.9 (3)	C8—C9—C14—O4A	−7.1 (5)
C3—C4—C5—C6	−2.0 (3)	C10—C9—C14—O4A	170.2 (3)
C3—C4—C5—S1	173.98 (14)	O6—C15—C16—C17	−179.34 (19)
C4—C5—C6—C7	1.5 (3)	O5—C15—C16—C17	0.0 (3)
S1—C5—C6—C7	−174.49 (16)	C15—C16—C17—C11	−2.1 (3)
C5—C6—C7—C2	0.3 (3)	C15—C16—C17—C18	177.28 (18)
C3—C2—C7—C6	−1.4 (3)	C10—C11—C17—C16	1.5 (2)
C1—C2—C7—C6	178.9 (2)	C12—C11—C17—C16	−178.16 (18)
C13—C8—C9—C10	−2.8 (3)	C10—C11—C17—C18	−177.90 (17)
O3—C8—C9—C10	179.68 (15)	C12—C11—C17—C18	2.5 (3)
C13—C8—C9—C14	174.6 (2)	C13—C8—O3—S1	80.5 (2)
O3—C8—C9—C14	−2.9 (3)	C9—C8—O3—S1	−101.90 (18)
C8—C9—C10—O5	−178.68 (15)	C11—C10—O5—C15	−3.4 (2)
C14—C9—C10—O5	3.9 (3)	C9—C10—O5—C15	177.47 (15)
C8—C9—C10—C11	2.2 (2)	O6—C15—O5—C10	−177.79 (16)
C14—C9—C10—C11	−175.20 (18)	C16—C15—O5—C10	2.8 (2)
O5—C10—C11—C12	−179.13 (16)	C8—O3—S1—O1	−175.87 (15)
C9—C10—C11—C12	−0.1 (3)	C8—O3—S1—O2	−48.35 (16)
O5—C10—C11—C17	1.2 (2)	C8—O3—S1—C5	67.94 (15)
C9—C10—C11—C17	−179.74 (15)	C4—C5—S1—O1	169.10 (15)
C10—C11—C12—C13	−1.7 (3)	C6—C5—S1—O1	−14.87 (19)
C17—C11—C12—C13	177.95 (18)	C4—C5—S1—O2	33.42 (17)
C11—C12—C13—C8	1.2 (3)	C6—C5—S1—O2	−150.56 (16)
C9—C8—C13—C12	1.2 (3)	C4—C5—S1—O3	−81.39 (16)
O3—C8—C13—C12	178.67 (18)	C6—C5—S1—O3	94.64 (16)

### Hydrogen-bond geometry (Å, °)

**Table:**

*D*—H···*A*	*D*—H	H···*A*	*D*···*A*	*D*—H···*A*
C4—H4···O6^i^	0.93	2.56	3.326 (2)	140
C13—H13···O6^ii^	0.93	2.52	3.343 (2)	148
C16—H16···O2^iii^	0.93	2.51	3.433 (2)	175

Symmetry codes: (i) −*x*+3/2, *y*+1/2, *z*; (ii) *x*−1/2, *y*, −*z*+3/2; (iii) *x*+1/2, *y*, −*z*+3/2.

## References

[bb1] Bruker (2001). *SMART* and *SAINT* Bruker AXS Inc., Madison, Wisconsin, USA.

[bb2] Carlton, B. D., Aubrun, J. C. & Simon, G. S. (1996). *Fundam. Appl. Toxicol.* **30**, 145–151. 10.1006/faat.1996.0051 8812259

[bb3] El-Agrody, A. M., Abd El-Latif, M. S., El-Hady, N. A., Fakery, A. H. & Bedair, A. H. (2001). *Molecules*, **6**, 519–527.

[bb4] Emmanuel-Giota, A. A., Fylaktakidou, K. C., Hadjipavlou-Litina, D. J., Litinas, K. E. & Nicolaides, D. N. (2001). *J. Heterocycl. Chem.* **38**, 717–722.

[bb5] Farrugia, L. J. (1999). *J. Appl. Cryst.* **32**, 837–838.

[bb6] Kalkhambkar, R.G., Kulkarni, G.M., Kamanavalli, Premkumar, N. Asdaq, S.M.B. & Sun, C.M. (2008). *Eur. J. Med. Chem.* **43**, 2178–2188. 10.1016/j.ejmech.2007.08.007 17959273

[bb7] Kulkarni, M. V., Kulkarni, G. M., Lin, C. H. & Sun, C. M. (2006). *Curr. Med. Chem.* **13**, 2795–2818. 10.2174/092986706778521968 17073630

[bb8] Nardelli, M. (1995). *J. Appl. Cryst.* **28**, 659.

[bb9] Shaker, R. M. (1996). *Pharmazie*, **51**, 148–148. 8900865

[bb10] Sheldrick, G. M. (2008). *Acta Cryst.* A**64**, 112–122. 10.1107/S0108767307043930 18156677

[bb11] Spek, A. L. (2009). *Acta Cryst.* D**65**, 148–155. 10.1107/S090744490804362X PMC2631630 19171970

[bb12] Yang, H., Protiva, P., Gil, R. R., Jiang, B., Baggett, S., Basile, M. J., Reynertson, K. A., Weinstein, I. B. & Kennelly, E. J. (2005). *Planta Med.* **71**, 852–60. 10.1055/s-2005-871257 16206041

[bb13] Yuvaraj, H., Gayathri, D., Kalkhambkar, R. G., Kulkarni, G. M. & Bapset, R. M. (2011). *Acta Cryst.* E**67**, o323. 10.1107/S1600536810054620 PMC3051468 21523009

[bb14] Zhou, P., Takaishi, Y. & Duan, H. (2000). *Phytochemistry*, **53**, 689–697. 10.1016/s0031-9422(99)00554-3 10746882

